# Modular Organization and Combinatorial Energetics of Proline–Tyrosine Nuclear Localization Signals

**DOI:** 10.1371/journal.pbio.0060137

**Published:** 2008-06-03

**Authors:** Katherine E Süel, Hongmei Gu, Yuh Min Chook

**Affiliations:** Department of Pharmacology, University of Texas Southwestern Medical Center at Dallas, Dallas, Texas, United States of America; The Rockefeller University, United States of America

## Abstract

Proline–tyrosine nuclear localization signals (PY-NLSs) are recognized and transported into the nucleus by human Karyopherin (Kap) β2/Transportin and yeast Kap104p. Multipartite PY-NLSs are highly diverse in sequence and structure, share a common C-terminal R/H/KX_2–5_PY motif, and can be subdivided into hydrophobic and basic subclasses based on loose N-terminal sequence motifs. PY-NLS variability is consistent with weak consensus motifs, but such diversity potentially renders comprehensive genome-scale searches intractable. Here, we use yeast Kap104p as a model system to understand the energetic organization of this NLS. First, we show that Kap104p substrates contain PY-NLSs, demonstrating their generality across eukaryotes. Previously reported Kapβ2–NLS structures explain Kap104p specificity for the basic PY-NLS. More importantly, thermodynamic analyses revealed physical properties that govern PY-NLS binding affinity: (1) PY-NLSs contain three energetically significant linear epitopes, (2) each epitope accommodates substantial sequence diversity, within defined limits, (3) the epitopes are energetically quasi-independent, and (4) a given linear epitope can contribute differently to total binding energy in different PY-NLSs, amplifying signal diversity through combinatorial mixing of energetically weak and strong motifs. The modular organization of the PY-NLS coupled with its combinatorial energetics lays a path to decode this diverse and evolvable signal for future comprehensive genome-scale identification of nuclear import substrates.

## Author Summary

To travel between the cytoplasm and nucleus, proteins rely on a family of transport proteins known as the karyopherinβ family. Karyopherinβ2, the human version of a family member, recognizes cargo proteins containing a class of nuclear localization signal known as the PY-NLS. The yeast homolog of Karyopherinβ2, Kap104p, also recognizes PY-NLSs, indicating that this pathway has been conserved between evolutionarily distant species. We mutated residues in the PY-NLSs of two Kap104p cargo proteins and analyzed how tightly these mutants bound Kap104p. These experiments revealed three PY-NLS regions, or epitopes, that are important for binding Kap104p. Each epitope is composed of amino acids that vary between cargoes. The epitopes are energetically independent and bind Kap104p with varying strengths in different PY-NLSs, such that mutating the epitope of one PY-NLS may mistakenly direct cargo to the cytoplasm, while a similar mutation in a different PY-NLS has little effect on cargo localization. This flexible, energetically modular, and combinatorial architecture of PY-NLSs may confer higher tolerance to mutations, but it also allows greater sequence diversity, making prediction of new PY-NLSs difficult. The characteristics of PY-NLSs reported here will assist in the identification of new Kap104p cargoes. And the approach used may be applicable to other biological recognition pathways.

## Abstract 3

PY-nuclear localization signals contain three binding regions that are not closely related in sequence and are energetically quasi-independent. These modular epitopes can contribute differently to the total binding energy in different signals, to tune their affinity for binding to the carrier protein Karyopherinβ2/Kap104p, and also to amplify signal diversity.

## Introduction

Karyopherinβ proteins (Kapβs; Importins/Exportins) mediate the majority of nucleocytoplasmic protein transport. There are 19 known Kapβs in human and 14 in yeast [1,2]. Kapβs bind substrates through nuclear localization or export signals (NLSs or NESs) and transport them through the nuclear pore complex, and Ran GTPase regulates Kapβ–substrate interactions [3–6]. Ten Kapβs are known to function in nuclear import, each recognizing at least one distinct NLS.

The best-known NLS is the short, basic, classical NLS, which is recognized by Kapα/Kapβ1 [4], and this pathway is conserved functionally from human to yeast [7,8]. Classical NLSs can be divided into monopartite and bipartite NLSs. Monopartite NLSs contain a single cluster of basic residues, whereas bipartite sequences contain two clusters of basic residues separated by a 10–12 amino acid linker. Thermodynamic dissection by scanning alanine mutagenesis of monopartite NLSs from the SV40 large T antigen (PKKKRKV) and the *c-myc* proto-oncogene (PAAKRVKLD) [9–11] confirmed a previously determined consensus sequence of K(K/R)X(K/R) [8,12]. Binding energies of these small signals are dominated by a single lysine residue, in the third position of the SV40 large T antigen and in the fourth position of c-myc, which makes numerous interactions with Kapα [9]. Thus, in the monopartite classical NLS, it is well-known that a relatively small motif is recognized, and binding energy is concentrated in stereotypical fashion across small sequences. Although numerous structures are available for bipartite NLSs [13–15], thorough thermodynamic analysis of this subclass is not available, and its consensus is less well-defined (one example is KRX_10–12_KRRK) than that for the monopartite NLS. Furthermore, a nonfunctional SV40 NLS mutant was rescued by a bipartite-like addition of a two-residue N-terminal basic cluster [9], suggesting that bipartite classical NLSs can accommodate larger sequence diversity than their monopartite counterparts.

Recently, structural and biochemical analyses of human Kapβ2 (Transportin) bound to the hnRNP A1 NLS revealed physical rules that describe Kapβ2′s recognition of a diverse set of 20–30-residue-long NLSs that we termed PY-NLSs [16]. These rules are structural disorder of a 30-residue or larger peptide segment, overall basic character, and weakly conserved sequence motifs composed of a loose N-terminal hydrophobic or basic motif and a C-terminal RX_2–5_PY motif. The composition of the N-terminal motifs divides PY-NLSs into hydrophobic and basic subclasses (hPY- and bPY-NLSs). The former contains four consecutive predominantly hydrophobic residues, while the equivalent region in bPY-NLSs is enriched in basic residues.

Approximately 100 different human proteins have been identified as potential Kapβ2 substrates [16–25]. Table 1 summarizes previously reported validated and potential PY-NLSs. Although many of these potential substrates were predicted by bioinformatics [16] and still need experimental testing, more than 20 have been validated for Kapβ2 binding (Table 1) [16–25]. Comparison of in vivo and in vitro validated PY-NLSs shows large sequence diversity, which is reflected in weak consensus sequences [16]. Structures of five different Kapβ2-bound PY-NLSs also show substantial variability, with structurally diverse linkers separating the convergent consensus regions [16,26,27]. The PY-NLS is significantly larger than the short monopartite classical NLS. The well-defined consensus and concentrated binding energy of the latter may reflect compactness of the signal. In contrast, the binding energy of the PY-NLS is spread over a much larger sequence. Physical properties of the multipartite PY-NLS may be more similar to those of the less-studied, larger, and sequentially more diverse bipartite classical NLS.

**Table 1:**
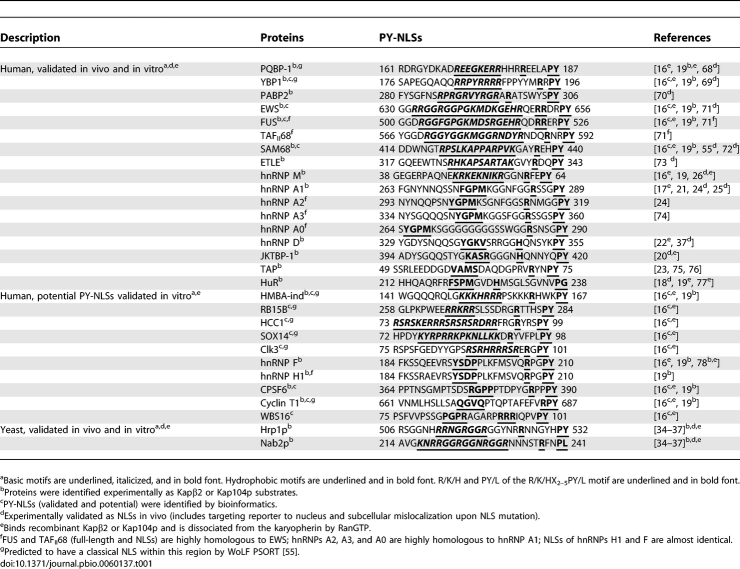
Summary of Validated and Potential PY-NLSs

Diverse PY-NLSs are described necessarily by weak consensus motifs. Therefore, instead of the traditional way of describing a linear recognition motif with a strongly restrictive consensus sequence, PY-NLSs were described by a collection of individually weak physical rules that together were able to provide substantial limits in sequence space for reasonable predictions of new Kapβ2 substrates [16]. However, the currently predicted substrates are most likely only a fraction of all PY-NLS-containing proteins because narrow sequence patterns were used in the initial search to achieve optimal accuracy. In fact, the sequence patterns used [16] were too narrow to predict PY-NLSs in known substrates HuR, TAP, hnRNP F, and JKTBP-1. The coverage of conventional sequence-based bioinformatics searches is expected to be severely limited due to PY-NLS diversity. Although sequence patterns obviously need to be expanded, we do not yet understand the limits of sequence diversity within motifs or how the different motifs may be combined. Knowledge of how binding energy is parsed in PY-NLSs will shape future efforts to decode these highly degenerate signals. Furthermore, physical understanding of how diverse PY-NLS sequences can achieve common biological function also will provide unique insights into many biological recognition processes that involve linear recognition motifs with weak and obscure consensus sequences, such as vesicular cargo sorting and protein targeting to the mitochondria and the peroxisome [28–33].

The yeast homolog of Kapβ2 is Kap104p (32% sequence identity) [34]. Only two Kap104p substrates, the mRNA processing proteins Nab2p and Hrp1p, are known. Several groups have mapped and validated NLSs of these substrates using both in vivo and in vitro methods to arginine–glycine (RG)-rich regions that were termed rg-NLSs [35–37]. Little sequence homology was detected between NLSs recognized by Kapβ2 and Kap104p. Furthermore, substrate recognition by the two karyopherins appears nonanalogous, as Kap104p does not recognize human substrate hnRNP A1 [35,37]. Given the recent physical understanding of Kapβ2–NLS interactions, we seek to examine the evolutionary conservation and energetic organization of signals in this pathway through studies of Kap104p–NLS interactions.

First, we present biochemical and biophysical analyses showing that RG-rich substrates of yeast Kap104p share similar physical characteristics to those of human PY-NLSs. Kap104p recognizes the basic but not hydrophobic PY-NLS subclass, and structural analyses of Kapβ2–NLS complexes suggested the origin of this specificity, enabling prediction of PY-NLS subclass specificity for all eukaryotic Kapβ2s. Thermodynamic analyses of Kap104p–NLS interactions revealed biophysical properties that govern binding affinity of PY-NLSs. These signals contain at least three energetically significant binding epitopes that are also linear motifs. Each linear epitope accommodates significant sequence diversity, and we have characterized some of the limits of this diversity. The linear epitopes are also energetically quasi-independent, a property that is probably due to intrinsic disorder of the free signals. Finally, in different PY-NLSs, a given epitope can vary significantly in its contribution to total binding energy. When combined with multivalency, this energetic variability can amplify signal diversity through combinatorial mixing of energetically weak and strong motifs.

## Results

### Yeast rg-NLSs Are Also PY-NLSs

**Figure 1:**
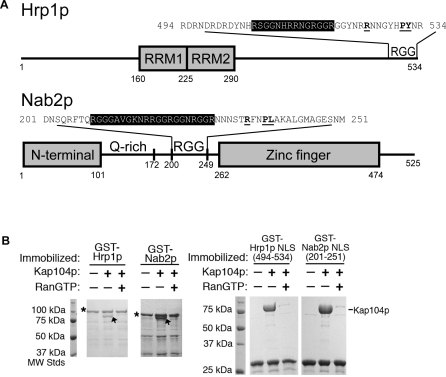
Interactions between Kap104p and Import Substrates Hrp1p and Nab2p (A) Domain organization of Hrp1p and Nab2p. Domains are indicated by gray boxes, and the RGG regions and the glutamine-rich region are labeled. The sequences of the two NLSs are shown, with the basic motif highlighted in black and the RX_2–5_PY(L) motif in bold and underlined. (B) Binding assays of Kap104p (arrow) with immobilized full-length Hrp1p and Nab2p (asterisks) or Hrp1p and Nab2p NLSs, in the presence and the absence of RanGTP. Bound proteins are stained with Coomassie blue.

In vivo validated RG-rich NLSs of Hrp1p and Nab2p (or rg-NLSs) are located at residues 494–534 and 201–250, respectively (Figure 1A) [35–38]. Examination of their sequences revealed physical characteristics similar to those of human PY-NLSs. Hrp1p and Nab2p NLSs are located within structurally disordered segments of 120–190 residues (DisEMBL structural disorder probabilities of 0.72 and 0.63 for Hrp1p and Nab2p, respectively [39]) in the full-length proteins (Figure 1A). ^506^ **R**SGGN**HRR**NG**R**GG**R** ^519^ of Hrp1p and ^216^ **K**N**RR**GG**R**GGN**R**GG**R** ^229^ of Nab2p contain many basic residues, like basic N-terminal motifs in human Kapβ2 substrates hnRNP M, PQBP-1, and YB-1 (Table 1) [16]. Closer to the C termini, the Hrp1p ^525^ **R**NNGYH**PY** ^532^ and the Nab2p ^235^ **R**FN**PL** ^239^ segments either match or are homologous to the C-terminal RX_2–5_PY consensus.

Immobilized full-length Hrp1p, Nab2p, and their NLSs bound Kap104p in stoichiometric proportions in pull-down binding assays (Figure 1B). Although it was previously reported that Ran could not dissociate substrate from Kap104p [36], we observed efficient dissociation of both full-length substrates and NLSs by RanGTP, possibly due to higher activity and GTP loading of the recombinant Ran. Our results suggest that Kap104p–NLS interactions and regulation by Ran are similar to other characterized Kapβ-mediated nuclear import processes in human [3–6]. Thermodynamic parameters for Kap104p binding to Hrp1p and Nab2p NLSs were obtained by isothermal titration calorimetry (ITC) (Figure S1). Both NLSs bound Kap104p with high affinity (*K*_D_ of 32 nM for Hrp1p and 37 nM for Nab2p (Tables 2 and 3)), and extensive mutagenesis of NLSs is discussed below. Thus, on the basis of their sequence characteristics, high affinity for karyopherin, and dissociation by RanGTP, yeast NLSs recognized by Kap104p resemble PY-NLSs.

**Table 2:**
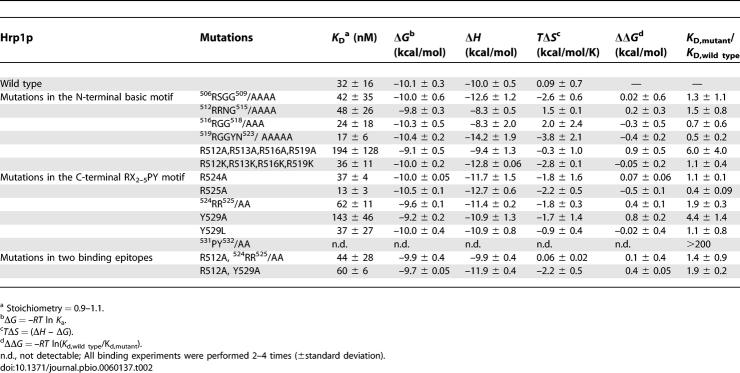
Summary of ITC Data for Kap104p Binding to Hrp1p Mutants

**Table 3:**
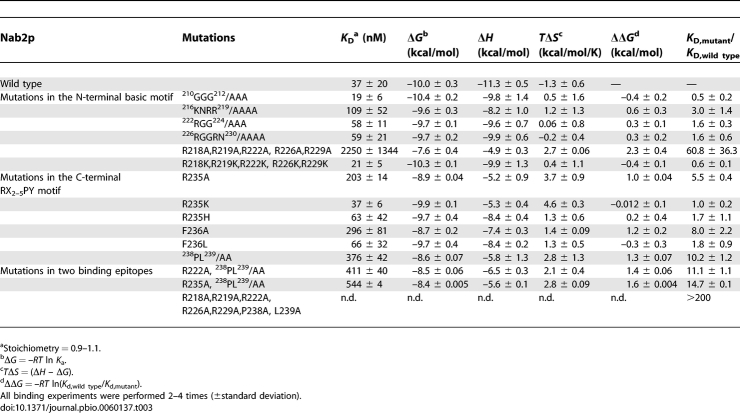
Summary of ITC Data for Kap104p Binding to Nab2p Mutants

### Kap104p Recognizes the Basic but Not Hydrophobic Subclass of PY-NLSs

**Figure 2:**
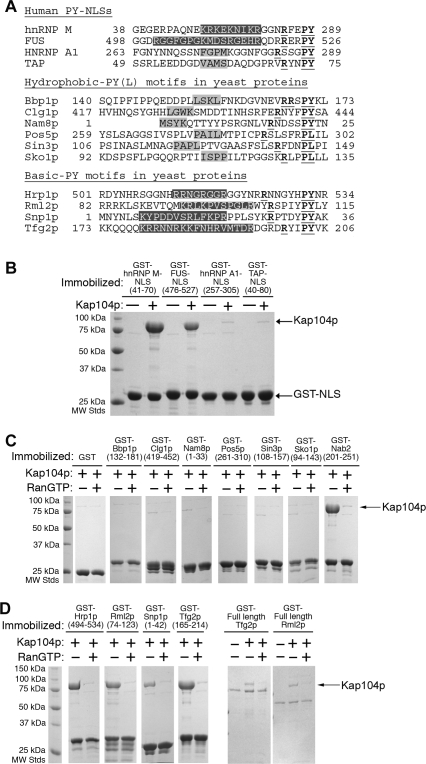
Kap104 Recognizes Basic PY-NLSs but Not Hydrophobic PY-NLSs (A) Sequences of known PY-NLSs in human proteins and predicted hydrophobic-PY(L) (shaded in light gray) and basic PY motifs (shaded in dark gray) in yeast proteins. The RX_2–5_PY(L) motif is underlined. (B) Binding assays of Kap104p and immobilized human bPY-NLSs (hnRNP M and FUS) or human hPY-NLSs (hnRNP A1 and TAP). (C) Binding assays of Kap104p with six immobilized predicted yeast hPY(L)-NLSs. GST and GST–Nab2p are included as controls. Faint bands at ∼70 kDa are likely heat shock protein contaminants. (D) Binding assays of Kap104p and immobilized predicted bPY-NLSs or full-length proteins. GST–Hrp1p is included as a control. Bound proteins are stained with Coomassie blue.

To investigate the PY-NLS subclass specificity of Kap104p, we examined its interaction with several human hPY- and bPY-NLSs as well as several predicted (see below) yeast hPY- and bPY-NLSs. Splicing factor hnRNP A1 and mRNA transport factor TAP/NXF1 contain hPY-NLSs, and splicing factor hnRNP M and FUS contain bPY-NLSs (Figure 2A). All four human PY-NLSs interacted with Kapβ2 [16], but only bPY-NLSs from hnRNP M and FUS bound yeast Kap104p in GST pull-down assays (Figure 2B). Both yeast Hrp1p and Nab2p NLSs bound equally well to Kap104p and Kapβ2 (Figure S2).

Hrp1p and Nab2p are the only two known Kap104p substrates [34–36]. We needed to identify additional yeast sequences to test the preference of Kap104p for bPY-NLS. Because Nab2p has a C-terminal PL instead of PY motif, suggesting that PL motifs also may be present in other functional PY-NLSs, we used the program ScanProsite [40] and sequence patterns Φ_1_-G/A/S-Φ_3_-Φ_4_-X_7–12_-R/K/H-X_2–5_-P-Y/L (where Φ_1_ is a hydrophobic residue and Φ_3_ and Φ_4_ are hydrophobic residues or R or K) [16] to search for potential hPY-NLSs within Saccharomyces cerevisiae proteins in the UniProtKB/Swiss-Prot protein database [41]. A consensus sequence for the N-terminal motif of bPY-NLSs is not available due to lack of an apparent specific pattern. As a result, we modified a previously used sequence pattern that is consistent with the basic motifs of hnRNP M and PQBP-1 [16] to accommodate additional validated human bPY-NLSs and NLSs in Nab2p and Hrp1p (Table 1). The resulting sequence pattern K/R-X_0–6_-K/R-X_0–6_-K/R-X_0–6_-K/R-X_2–5_-R/K/H-X_1–5_-PY/L is used to search for potential yeast bPY-NLSs. The resulting lists were filtered for structural disorder [39] and overall basic character. Six hPY/L-containing fragments were tested, but none bound Kap104p (Figure 2A and 2C). However, 11 of 20 bPY/L-containing fragments tested bound Kap104p and were dissociated by RanGTP (Figure 2A and 2D and Figure S3a and S3b). Two bPY/L-containing full-length substrates, Tfg2p and Rml2p, were tested, and both bound Kap104p and were dissociated by RanGTP (Figure 2D). Of the 11 bPY/L-containing proteins in yeast that bound Kap104p (Table 1), 7 (or 64%) have been shown to be predominantly nuclear or show both nuclear and cytoplasmic localization. Thus, recognition of the basic subclass of PY-NLS is conserved between human and yeast. However, human Kapβ2 has evolved to recognize an additional hydrophobic PY-NLS subclass, enabling it to transport a broader range of substrates. Alternatively, Kap104p may have evolved to be more specific and lost its ability recognize hPY-NLSs.

### Kapβ2-NLS Structures Explain Kap104p Subclass Specificity

**Figure 3:**
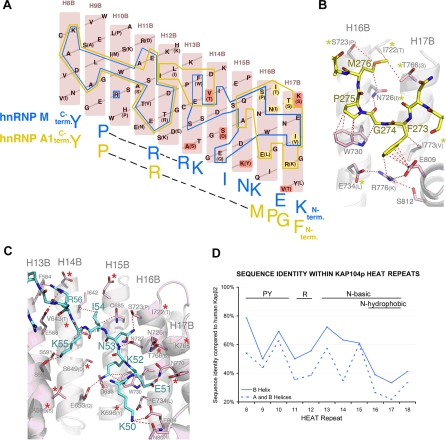
Kapβ2–NLS Structures and Kap104p Specificity (A) Schematic representation of the Kapβ2–NLS interface showing B helices of Kapβ2 H8–H17 (pink). Residues that are different in Kap104p are in parentheses. Kapβ2 residues that contact the hnRNP A1 NLS (hPY-NLS) and the hnRNP M NLS (bPY-NLS) are outlined in yellow and blue, respectively. Residues contacting the N-terminal FGPM hydrophobic motif in hnRNP A1, which are also different in Kap104p, are highlighted in yellow. Residues that increase electronegativity of the Kap104p surface are highlighted in red. (B) Interactions between Kapβ2 (pink) and the N-terminal hydrophobic motif of hnRNP A1 (yellow) (PDB ID 2H4M) drawn with PYMOL [67]. Residues that are different in Kap104p are in parentheses (yellow asterisks label residues that may affect interactions with hPY-NLSs). (C) Interactions between Kapβ2 (pink) and the N-terminal basic motif of hnRNP M (blue) (PDB ID 2OT8). Residues that are different in Kap104p are in parentheses. Red asterisks label Kap104p substitutions that increase electronegativity. (D) Sequence identity within individual HEAT repeats of Kapβ2 and Kap104p. The motifs recognized by the B helix of each HEAT repeat are specified above the graph.

Kapβ2 and Kap104p sequences were aligned and examined in the context of crystal structures of Kapβ2 bound to NLSs of hnRNPs A1 (hPY-NLS) and M (bPY-NLS) [16,26]. Kapβ2 has 20 HEAT repeats, each consisting of two antiparallel helices A and B. Both PY-NLSs bind the Kapβ2 interface lined with B helices of HEAT repeats 8–18 (abbreviated H8B–H18B), converging structurally at three spatially distinct binding sites: (1) overlapping portions of the N-terminal hydrophobic and the larger basic motifs, (2) the arginine residue, and (3) the PY residues, both of the C-terminal RX_2–5_PY motifs [26]. Correspondingly, both structures share many common Kapβ2 interface residues, especially those that contact the conserved C-terminal RX_2–5_PY motif (Figure 3A).

Approximately half of the Kapβ2–NLS interface residues are conserved in Kap104p. Interfaces with the RX_2–5_PY motifs (H8B–H12B) are mostly invariant, while differences occur at the structurally overlapping interfaces with the basic/hydrophobic N-terminal motifs (H15B–H17B) and at linker regions (H12B–H14B) (Figure 3A). Here, Kapβ2 residues I722, S723, N726, E734, T766, and I773 that contact the hnRNP A1 hydrophobic motif are replaced with T, P, I, L, S, and V, respectively, in yeast (Figure 3A and 3B) such that many hydrophobic contacts with the FGPM N-terminal motif of hnRNP A1 are expected to be lost in yeast (detailed description in Text S1). In contrast, among Kapβ2 residues that contact basic side chains of bPY-NLSs, only E653 of Kapβ2 is different in yeast (Figure 3A and 3C), and several amino acids have been replaced by more electronegative amino acids in Kap104p (Figure 3C), further supporting bPY-NLS recognition in yeast.

Comparison of individual HEAT repeats of Kapβ2 and Kap104p showed high identity (∼50%) at H8–H10, but the similarity dropped to ∼20% at H17 (Figure 3D). The B helices that line the interface are generally more conserved than the outer A helices. However, even in the former, sequence identities in H16B–H17B dipped significantly below 40% (Figure 3D). These observations suggest that both helical orientations and interface functional groups are better conserved at recognition sites for the C-terminal PY motif (H8–H10) than at the N-terminal basic/hydrophobic motifs (H16–H17). Consequently, the loss of Kap104p recognition for the N-terminal hydrophobic motif is most likely due to critical interface residue changes in H16B–H17B and to changes in helical orientations in this region. We have aligned sequences of Kapβ2 homologs, tracked interface residues and potential overall helical similarities at the N-terminal hydrophobic motif interfaces in different organisms, and used this information to predict species in which Kapβ2 would recognize hPY-NLSs. Results of these studies are discussed in Text S2 and shown in Figure S4A and S4B.

### Distribution of Binding Energy along the Hrp1p NLS

**Figure 4:**
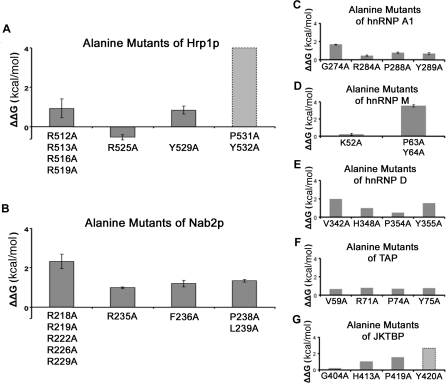
Mutagenic Analyses of the Hrp1p NLS, Nab2p NLS, and Human PY-NLSs (A and B) Loss of Kap104p binding energy in alanine mutants of (A) Hrp1p and (B) Nab2p (ΔΔ*G* = –*RT* ln(*K*_D,wild type_/*K*_D,mutant_). (C–G) Loss of Kapβ2 binding energy in alanine mutants of PY-NLSs from (C) hnRNP A1, (D) hnRNP M, (E) hnRNP D, (F) TAP, and (G) JKTBP (ΔΔ*G* = –*RT* ln(*K*_D,wild type_/*K*_D,mutant_). *K*_D,wild type_ and *K*_D,mutant_ values for hnRNP A1 were obtained from [16], hnRNP M from [26], hnRNP D, TAP, and JKTBP from [27]. *K*_D_ values for Hrp1p, Nab2p, and hnRNPs A1 and M were obtained by ITC whereas those for hnRNP D, TAP, and JKTBP were obtained by surface plasmon resonance.

We have performed scanning alanine mutagenesis covering residues 506–532 of the Hrp1p NLS (Figure 1A, Table 2, and Table S1). In the N-terminal region of the Hrp1p NLS, none of the four mutants ^506^RSGG^509^/AAAA, ^512^RRNG^515^/AAAA, ^516^RGG^518^/AAA, and ^519^RGGYN^523^/AAAAA (Table 2) affected Kap104p binding, suggesting that this N-terminal basic-enriched region may contribute little to total binding energy. However, these mutations may be misleading as glycine to alanine mutations may decrease the entropy of the unbound NLS, thus decreasing the entropic penalty of binding and offsetting affinity loss from arginine mutations. Therefore, we also generated a quadruple mutant where all of the arginines (R512, R513, R516, and R519) were mutated to alanines. This quadruple mutant decreased Kap104p binding by a marginal 5-fold (Figure 4A and Table 2), suggesting that positive charges in the N-terminal basic region are somewhat important for Kapβ–NLS interaction. Quadruple mutant R512, R513, R516, R519/KKKK did not affect Kap104p binding (Table 2), further suggesting that stereospecific interactions with arginine guanido groups are not important for Kap104p binding.

Kap104p binding was not affected significantly when both arginine residues, ^524^RR^525^, in the C-terminal RX_2–5_PY motif of the Hrp1p NLS were mutated to alanines (*K*_D,mutant_/*K*_D,wild type_ = 1.7; Figure 4A and Table 2). In contrast, the C-terminal ^531^PY^532^/AA mutation abolished detectable Kap104p binding (Table 2). The enthalpies of binding for all of the PY-NLSs that we have measured by ITC are similar, and the weakest measurable *K*_D_ in this series was 10 μM [26]. Therefore, we assume that the affinity of the Hrp1p ^531^PY^532^/AA mutant is likely weaker than 10 μM and its *K*_D,mutant_/*K*_D,wild type_ > 200 (Figure 4A). Thus, the Hrp1p NLS contains one strong binding hotspot at its PY motif, similar to the single significant hotspot at the C-terminal PY motif of the human substrate hnRNP M (*K*_D,mutantPY/AA_/*K*_D,wild type_ = 500 for the hnRNP M NLS) [26]. Interestingly, we also located a modest binding hotspot at residue Y529 (*K*_D,mutant_/*K*_D,wild type_ = 4 for Y529A; Figure 4A and Table 2) in the linker between the arginine and the PY of the RX_2–5_PY C-terminal motif. However, the Y529L mutation did not affect Kap104p binding (Table 2), suggesting that a hydrophobic, but not necessarily aromatic, moiety at this position might be important.

### Distribution of Binding Energy along the Nab2p NLS

We have performed scanning alanine mutagenesis covering residues 210–239 of the Nab2p NLS (Figure 1A, Table 3, and Table S1). Binding energy along the Nab2p NLS appears quite distributed compared to that of the Hrp1p NLS, with no single binding hotspot that stands out above others (Figure 4B and Table 3). In its basic N-terminal region, ^216^KNRR^219^, ^222^RGG^224^, and ^226^RGGRN^230^ each were mutated to alanines, but only ^216^KNRR^219^/AAAA showed a small 3-fold decrease in Kap104p affinity (Table 3). None of the single mutants K216A, R218A, R219A, R222A, R226A, or R229A decreased Kap104p binding (Table S1), and simultaneous mutation of all of the arginines to lysines also did not decrease Kap104p binding. In contrast, mutation of all five arginines to alanines decreased affinity by 60-fold (*K*_D_ = 2.25 μM; Figure 4B and Table 3), suggesting that the collective basic character of this region contributes significantly to the total binding energy of the NLS. Comparison of single arginine to alanine mutants (*K*_D,mutant_/*K*_D,wild type_ ≈ 1.0) to the pentamutant R218, R219, R222, R226, R229/AAAAA (*K*_D,mutant_/*K*_D,wild type_ = 60.8) indicated a binding cooperativity of at least 60-fold within the N-terminal basic motif of Nab2p.

When R235 of the Nab2p C-terminal RX_2–5_PL motif was mutated to an alanine, Kap104p affinity decreased by 5-fold (Figure 4B and Table 3). Crystal structures of Kapβ2 bound to NLSs of hnRNPs A1 and M showed the equivalent arginine residues making electrostatic interactions with numerous aspartate and glutamate residues, suggesting the importance of a positively charged residue at this position [16,26]. We also mutated R235 to lysine and histidine, but neither mutant affected Kap104p binding significantly (*K*_D,mutant_/*K*_D,wild type_ are 1.0 and 1.7, respectively; Table 3). The C-terminal ^238^PL^239^/AA mutation in the Nab2p NLS decreased Kap104p binding by 10-fold (Figure 4B and Table 3). The energetic significance of this mutation suggests its equivalence to the PY motif in human Kapβ2 substrates and in Hrp1p. Furthermore, the Nab2p ^238^PL^239^/PY mutant bound Kap104p with a slightly higher affinity at a *K*_D_ value of 13 nM. Mutagenesis of residue L239 to all other amino acids is described below.

The measurable ^238^PL^239^/AA mutation in the Nab2p NLS (*K*_D_ = 376 nM) provided an opportunity to explore cooperativity across binding sites or epitopes. Mutations in the Nab2p triple mutant R222A, ^238^PL^239^/AA (*K*_D_ 411 nM; *K*_D,mutant_/*K*_D,wild type_ = 11.1; Table 3) show almost perfect additivity when compared to a single R222A mutant (did not affect Kap104p binding; Table S1) and double mutant ^238^PL^239^/AA (*K*_D,mutant_/*K*_D,wild type_ = 10.2; Table 3). A second Nab2p triple mutant R235A, ^238^PL^239^/AA (*K*_D_ = 544 nM; *K*_D,mutant_/*K*_D,wild type_ = 14.7; Table 3) also was compared to a single R235A mutant (*K*_D,mutant_/*K*_D,wild type_ = 5.5; Table S1) and double ^238^PL^239^/AA mutant (*K*_D,mutant_/*K*_D,wild type_ = 10.2; Table 3). Strict additivity between the R and the PL sites would give a calculated *K*_D,mutant_/*K*_D,wild type_ value of 56.1 for the triple mutant. Thus, the experimental *K*_D,mutant_/*K*_D,wild type_ value of 14.7 for the triple mutant indicated 3.8-fold cooperativity between the two epitopes. Similarly, Hrp1p triple mutant R512A,^ 524^RR^525^/AA and double mutant R512A, Y529A showed cooperativity of approximately 1.4- and 2-fold between epitopes, respectively. The couplings between binding epitopes observed here for both Nab2p and Hrp1p are still more than an order of magnitude lower than that observed within the N-terminal basic region of Nab2p (>60-fold cooperativity).

We also located a new binding hotspot at F236 in Nab2p (*K*_D,mutant_/*K*_D,wild type_ = 8 for F236A; Figure 4B and Table 3), which is located in the linker between the R and the PL of the RX_2–5_PL C-terminal motif. This site is analogous to Y529 of Hrp1p discussed in the previous section, and both residues are located two residues N-terminal of the PY/L motifs. As in the Hrp1p NLS Y529L mutant, the F236L mutation in Nab2p did not affect Kap104p binding (Table 3). Aromatic or hydrophobic residues occur at this position in many human PY-NLSs, including hnRNPs M, D, and F, JKTBP, TAP, HMBA-inducible protein, PABP2, PQBP-1, RB15B, and WBS-16 [16,22,23,27]. Aromatic side chains at this position overlap in the crystal structures of Kapβ2 bound to the NLSs of hnRNPs M and D and TAP [26,27]. The F61 of the hnRNP M NLS, Y352 of the hnRNP D NLS, and Y72 of the TAP NLS make hydrophobic interactions with Kapβ2 W460A and with the backbones of the PY motifs. A hydrophobic residue here may contribute to binding energy through both favorable enthalpy and a decrease of entropic penalty upon binding by preorganizing the PY motif. Thus, if present, a hydrophobic residue here may be considered as an extension of the PY motif.

Hrp1p contains a single very significant binding hotspot at its PY motif. In contrast, binding energy in Nab2p is more evenly distributed across its N-terminal basic region and the R, F, and PL residues of its C-terminal consensus motif. Thus, distributions of binding energy in the two yeast NLSs are very different. From the N to C terminus, energetic distribution across the three epitopes (N-terminal basic region, R, and PY/L of the C-terminal motif) of Hrp1p and Nab2p can be described roughly as medium–weak–strong and strong–medium–medium, respectively (ΔΔ*G* < 0.9 kcal/mol is categorized as weak, 0.9 ≤ ΔΔ*G* ≤ 1.7 kcal/mol as medium, and ΔΔ*G* > 1.7 kcal/mol as strong; Figure 4A and 4B). Similarly, in previously characterized PY-NLSs of hnRNPs A1 and D, TAP, and JKTBP [16,27], energetic distributions at the three epitopes also are quite varied, with rough patterns of strong–weak–weak, strong–medium–medium, weak–weak–weak, and weak–medium–strong, respectively (Figure 4C–G). In summary, all three PY-NLS epitopes are energetically highly variable, the N-terminal basic/hydrophobic and the C-terminal PY motifs appear to cover the entire energetic continuum from strong to weak, and the arginine of the RX_2–5_PY motif is medium to weakly energetically significant.

#### Degeneracy of Tyrosine in the C-Terminal PY Motif

**Figure 5:**
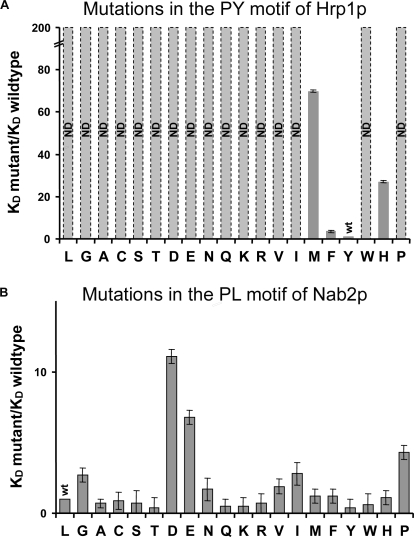
Mutagenic Analysis of the PY(L) Motif (A) Mutations in the PY motif of Hrp1p and the resulting fold decrease in binding affinity for Kap104p. Only M, F, and H can substitute for the Y. All other mutations had no detectable (ND) binding by ITC. (B) Mutations in the PL motif of Nab2p and the resulting fold decrease in binding affinity for Kap104p.

Of the more than 20 sequences that bind Kapβ2 and Kap104p (Table 1) [16], two do not contain the PY dipeptide in their C termini. HuR has a PG, and Nab2p has a PL, thus raising the question of degeneracy at this C-terminal position. We mutated Y532 in the PY motif of Hrp1p to the other 19 amino acids (Figure 5A and Table S2). Only Y532F, Y532H, and Y532M showed measurable Kap104p binding by ITC. Y532F best resembles the wild type, with only a 4-fold decrease in Kap104p affinity. Both Y532H and Y532M in Hrp1p bound significantly weaker with *K*_D_ values of 1 and 2 μM, respectively.

We also mutated L239 in the Nab2p PL motif to the other 19 amino acids (Figure 5B and Table S3). Binding energy along the Nab2p NLS is distributed very evenly compared to that of the Hr1p1p NLS with the Nab2p ^238^PL^239^/AA mutation decreasing affinity only 10-fold compared to the >200-fold effect in Hrp1p. Thus, in the energetically distributed Nab2p NLS, changes in the L239 position may be quite permissive. This is indeed the case because only L239D and L239E showed significant affinity decreases of 11- and 7-fold, respectively. L239G, L239I, and L230P showed a modest 3–4-fold affinity decrease. None of the other mutants (to S, T, N, Q, K, R, V, M, F, Y, W, and H) decreased Kap104p binding.

Tyrosine is clearly the most preferred residue in the last position of the Hr1p1 NLS. Correspondingly, mutation of the PL motif in Nab2p to PY improves Kap104p binding. These results suggest that, in general, tyrosine may be the most preferred and thus likely the most prevalent amino acid found in the last position of PY-NLSs (Table 1). It appears that if the PY site is energetically very significant, such as that in Hrp1p, the residue type allowed at the terminal position is quite restrictive, with only 2–4 residues (Y, F, H, and M) allowed. However, when the same motif is fairly silent energetically, such as that in Nab2p and hnRNP A1 [16], the distribution of allowed amino acids in the terminal position is likely much wider, with only 2–5 residues disallowed.

### Hrp1p and Nab2p Mutants Are Mislocalized In Vivo

**Figure 6:**
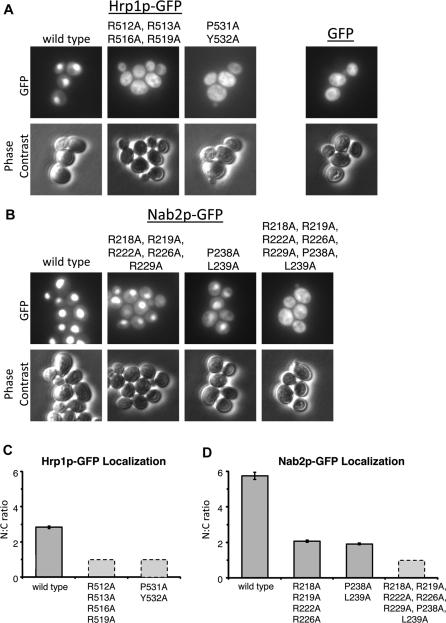
Hrp1p and Nab2p Mutants Are Mislocalized In Vivo (A) S. cerevisiae cells expressing either wild-type or mutant full-length Hrp1p–GFP fusion proteins were analyzed by fluorescence microscopy and phase contrast. GFP is displayed in the same fluorescence scale in each panel. (B) Cells expressing either wild-type or mutant full-length Nab2p–GFP fusion proteins were analyzed as in (A). (C and D) Mean pixel values were used to determine the nuclear/cytoplasmic (N/C) ratio of fluorescence intensity for either (C) Hrp1p–GFP or (D) Nab2p–GFP fusion proteins (±standard error of the mean). Dashed lines indicate an estimated N/C ratio of 1:1 due to the diffuse nuclear and cytoplasmic localization of the fusion protein.

To examine the effect of PY-NLS mutations on nucleocytoplasmic localization of Hrp1p and Nab2p in vivo, we expressed GFP-tagged full-length Hrp1p and Nab2p wild-type and mutant proteins in yeast. Wild-type Hrp1–GFP and Nab2p–GFP are localized in the nucleus as has been reported previously (Figure 6A–D) [34–36]. Mutations in the C-terminal PY motif (^531^PY^532^/AA) of Hrp1p, which abolished detectable Kap104p binding, resulted in mislocalization of the GFP fusion protein to the cytoplasm (Figure 6A and 6C). The N-terminal basic motif of Hrp1p is also important for nuclear localization of Hrp1p: the R512,R513,R516,R519/AAAA mutant, which decreased Kap104p binding by a marginal 5-fold, also is mislocalized (Figure 6A and 6C). Xu and Henry have shown previously that substitutions of R516 and R519 with glutamines mislocalized Hrp1p, but proteins with lysine substitutions are properly localized [38,42]. This further suggests that basic charges rather than stereospecific interactions are necessary for Kap104p interactions.

In the case of Nab2p, mutations in either the N-terminal motif (pentamutant R218,R219,R222,R226,R229/AAAAA; decreases Kap104p binding by 60-fold) or the C-terminal PY motif (^238^PL^239^/AA; decreases Kap104p binding by 10-fold) resulted in increased cytoplasmic localization of the GFP fusion protein (Figure 6B and 6D). Arginine methylation of Nab2p by Hmt1p is required for its export from the nucleus, possibly explaining some nuclear accumulation of the N-terminal mutant despite its low affinity for Kap104p [38,43]. Combined mutations of both the N- and the C-terminal motifs resulted in diffuse localization of the fusion protein, consistent with further affinity reduction for Kap104p (Table 3 and Figure 6B and 6D). We have shown here that mutations in the PY-NLSs of Hrp1p and Nab2p that decrease binding affinity to Kap104 also affect nuclear localization in yeast cells.

## Discussion

The problem of deciphering the sequence code for substrate recognition by Kapβ2 is interesting and challenging because the transport factor exhibits obvious biologically relevant specificity for nuclear import substrates but at the same time is able to handle a large number of different sequence-diverse substrates. Previous studies have captured the requirement for structural disorder in NLSs and the notion of a few anchoring amino acids such as the N-terminal hydrophobic/basic and RX_2–5_PY motifs [16,26]. Here, we show that yeast Kap104p is a PY-NLS-recognizing homolog specific for the basic subclass of this signal and that the two different Kap104p substrates have rather different distributions of binding energy for Kap104p. The NLS in Hrp1p largely uses the PY motif, and the NLS in Nab2p uses many positions distributed across three binding regions. Consistent with this, the Y position of the PY motif shows more degeneracy in Nab2p than in Hrp1p. On the basis of all of this and the thermodynamic data from five human PY-NLSs [16,26,27], we propose the following physical properties that govern the affinity of PY-NLS recognition by Kapβ2:

### 1. PY-NLSs Contain at Least Three Energetically Significant Binding Epitopes

Structures of PY-NLSs from hnRNPs A1, M, and D, TAP, and JKTBP converge spatially at three distinct binding sites or epitopes separated by structurally variable linkers: (1) the N-terminal hydrophobic/basic motif, (2) the arginine residue of the C-terminal RX_2–5_PY sequence motif, and (3) the PY of the C-terminal RX_2–5_PY motif [16,26,27]. We have shown here that all three structural epitopes can be energetically significant.

The N-terminal basic-enriched motifs of Hrp1p and Nab2p NLSs constitute epitope 1, where collective basic character and likely charge density drive Kap104p binding. Mutations of all of the arginines in this region to alanines decreased binding energy by 0.9–2.3 kcal/mol for both NLSs. Similarly, the N-terminal hydrophobic motif of the hnRNP A1 NLS and the equivalent region of the hnRNP D NLS that contains both hydrophobic and basic residues are also energetically significant, with mutations decreasing binding energy by ∼2 kcal/mol [26].

Epitopes 2 and 3 are contained within the C-terminal RX_2–5_PY/L sequence motifs. Two linkers of variable lengths, compositions, and structures connect epitope 1 to epitope 2 and epitope 2 to epitope 3 [16,26]. Epitope 2 is located at Hrp1p ^524^RR^525^ and Nab2p R235 at the first consensus position of the C-terminal RX_2–5_PY/L sequence motifs. Of the three PY-NLS epitopes, epitope 2 tends to contribute the least to binding energy, with mutations decreasing binding energy maximally by ∼1 kcal/mol in Nab2p, hnRNP D, and JKTBP (Figure 4B, 4E, and 4G).

Epitope 3 is located at Hrp1p ^531^PY^532^ and Nab2p ^238^PL^239^. Mutations at these terminal positions are generally energetically significant, decreasing binding energy by 1.3–4 kcal/mol in Hrp1p, Nab2p, hnRNPs M and D, and JKTBP. However, exceptions are seen in hnRNP A1 and TAP, where PY mutations decreased binding modestly by only ∼0.7 kcal/mol.

Because free PY-NLSs are structurally disordered and adopt extended Kapβ2-bound conformations, epitopes 1–3 are presented as peptides that can be represented by sequence patterns or linear motifs [44–46]. In epitope 1, the N-terminal basic motif may be represented by a collection of sequence patterns covering 5–19 residues, and the N-terminal hydrophobic motif by sequence patterns of approximately 4 residues. Epitopes 2 and 3 are both relatively smaller and simpler and together can be described by a single sequence pattern.

### 2. Each Linear Epitope Can Accommodate Large Sequence Diversity

Comparison of validated and potential PY-NLSs in Table 1 [16,26] show that sequences within each of the three linear epitopes can be quite variable. The N-terminal basic/hydrophobic motif is the largest and most variable epitope. Mutagenesis of yeast PY-NLSs has provided more information on the diversity and also suggested some limits to the diversity of individual epitopes. In particular, positive charges within the N-terminal basic motifs are important, but arginine and lysine residues are interchangeable, and the exact positions of basic groups may not be important (Tables 2 and 3 and Table S1). Additional biochemical and structural studies will be needed to understand requirements of charge density, segment size, and negatively selected amino acids in this epitope. The consensus for this basic region remains elusive. The 55% accuracy for bioinformatics-derived potential yeast bPY-NLSs binding to Kap104p may reflect high sequence variability and undiscovered physical characteristics of this region.

Epitope 2 is usually composed of a single residue. Examination of validated PY-NLSs (Table 1) shows that arginine is most prevalent in this position, although histidines are found in this position in hnRNP D, JKTBP, and HuR and lysines in potential yeast NLSs of Naf1p, Sbp1p, Arp8p, and Ste20p (Figure S3A). Mutagenesis has shown that arginine, lysine, and histidines are interchangeable in this position. Thus, the appropriate sequence pattern here is R/K/H.

Human Kapβ2 substrate HuR (Table 1) has a PG dipeptide, and yeast Nab2p and eight bioinformatics-derived potential yeast NLSs contain PL dipeptides at the C-terminal positions of their NLSs (epitope 3). In some cases, epitope 3 matters energetically more than in others. It is unclear why the dipeptide motif is energetically significant in some peptides and relatively silent in others. We speculate that a hydrophobic amino acid two residues N-terminal of the PY motif may be necessary (though probably not sufficient) and should be included in the sequence pattern for an energetically strong epitope 3. A hydrophobic residue at this position may preorganize the short peptide segment for binding, lowering both strain and entropic penalties. We also note that if epitope 3 is energetically very significant, then the terminal site tends to be phenylalanine, histidine, and methionine. If the dipeptide motif is fairly silent energetically, then many other amino acids are allowed in the terminal position.

### 3. Energetic Cooperativity Observed within Linear Epitopes but Not between Them

Mutations within a linear epitope such as within the N-terminal basic region of Nab2p show large cooperativity of >60-fold (Table 2 and Table S1). Mutations within the N-terminal basic region of the hnRNP M NLS also show cooperativity, in a similar regime, of ∼40-fold [26]. In contrast, seven examples of simultaneous mutations between different linear epitopes in Hrp1p, Nab2p (Tables 2 and 3), and hnRNPs A1 and M [16,26] show only modest cooperativities of 1.0–3.8-fold. Cooperativity between linear epitopes in PY-NLSs is also very small compared to that typically observed between spatially distinct sites in conformational epitopes. For example, in the interaction of human growth hormone with human growth hormone receptor, mutations at distant sites in the interface showed large cooperativity of ∼60-fold [47]. Thus, by comparison, the linear epitopes in PY-NLSs are energetically quasi-independent. In an analogous system, a bipartite interaction in a linear sorting signal in a SNARE and COPII coat also exhibited energetic quasi-independence, showing only a 1.5–2-fold cooperative effect between the two distant sites [48]. In both PY-NLSs and vesicular sorting signals, minimal coupling between linear epitopes, and thus energetic modularity of those epitopes, may be attributed to flexible or structurally variable linkers that connect the epitopes.

### 4. Energetically Variable Linear Epitopes Can Be Mixed in a Combinatorial Fashion

Finally, the fourth biophysical property that governs PY-NLS affinity stems from the observation that binding energy is distributed very differently amongst the three linear epitopes in all seven thermodynamically characterized PY-NLSs [16,26,27]. In different PY-NLSs, a given linear epitope can vary significantly in its contribution to total binding energy. For example, the N-terminal basic motif in Hrp1p contributes much less to Kap104p binding than the equivalent epitope in Nab2p (compare Figure 4A and 4B). Similarly, PY in hnRNP A1 contributes only weakly to Kapβ2 binding, while PY motifs in hnRNP M and Hrp1p are the sole binding hotspots in the NLSs (Figure 4A, 4C, and 4D). We previously had taken advantage of the energetic variability of PY-NLS epitopes by harnessing the avidity effect of the NLS hotspot at epitope 1 of hnRNP A1 fused to the NLS hoptspot at epitope 3 of hnRNP M, which resulted in a chimeric peptide inhibitor that bound Kapβ2 200-fold tighter than both substrates and RanGTP [26]. Despite the wide energetic variability of individual linear epitopes, the total binding energies are very similar for various PY-NLS-containing substrates. Therefore, evolution has not combined epitopes randomly but rather tuned them to a range for appreciable Kapβ2 binding and efficient Ran dissociation. The extremely tight-binding chimeric peptide inhibitor of Kapβ2 [26] is evidence of such evolutionary pressure. Although very high affinity can be achieved easily, nuclear import function is lost as RanGTP can no longer dissociate substrates.

Binding energy in the PY-NLS is distributed over a large sequence, with three different elements contributing differently in various substrates. It is this feature that makes the PY-NLS fundamentally different from the well-known monopartite classical NLS. A relatively small motif is recognized in a monopartite NLS, and binding energy is concentrated in a stereotypical fashion across small sequences.

### Modular and Combinatorial Design of PY-NLS May Be Highly Evolvable

In PY-NLSs, the three distinct linear sequence elements are presented on peptides that exhibit intrinsic structural disorder and bind Kapβ2 with extended structurally diverse conformations. This modular and flexible display of multiple sequence motifs is relatively free of spatial constraints that usually relate multiple binding sites within a folded ligand. Furthermore, when binding energy is variably distributed among multiple epitopes in PY-NLSs, single mutations or mutations within single NLS epitopes are likely to have decreased chances of abolishing karyopherin binding. Thus, the modular, flexible, and energetically combinatorial architecture of PY-NLSs may allow significant evolvability to form new interactions while maintaining Kapβ2 recognition. Similar “multifaceted” interactions, where different ligands make energetically significant interactions with different subsets of interface residues, were recently studied in a theoretical context [49] and also suggested to be more tolerant to mutations and are therefore quite evolvable.

Multiple functions have been identified in fact in several PY-NLSs. In Nab2p, the RGG region that overlaps NLS epitope 1 is a putative RNA binding region [50]. The PY-NLSs in Nab2p, Hrp1p, EWS, and FUS interact with and are methylated by arginine methyltransferases [43,51–54]. Phosphorylation sites also have evolved within PY-NLSs to regulate nucleocytoplasmic localization. Serine phosphorylation in the hnRNP A2 NLS and tyrosine phosphorylation in the SAM68 NLS [55] both alter subcellular localization of the proteins. A PY-NLS also may evolve additional NLSs within its sequence. This could generate redundancy in nuclear import pathways and also provide a path to switch substrates from one karyopherin to another and ultimately from one cellular process to another. We have identified a potential classical NLS [56] in the N-terminal basic motifs of eight human bPY-NLSs in Table 1. It is not clear what overlapping NLSs mean in the cellular context, but this question will need to be explored in the future.

### Path to Comprehensive PY-NLS Identification in Genomes

Identifying correct sequences that will account for most of the very diverse PY-NLS is an extremely challenging task. The core problem is that binding energy is distributed across three epitopes or motifs in many different ways. Thus, simply relaxing sequence constraints in a global search will also increase “noise” and result in many wrong answers.

We predict that if a PY motif (epitope 3) is energetically very significant, then the sequence tolerance for this motif is small, and sequence content of the other two epitopes will likely not matter. Thus, this subset of the PY-NLSs should be identified easily upon identification of PY motifs that can provide large binding energies. Given the relatively small size of this motif, the task of finding strong PY motifs should be experimentally accessible. A similar situation should apply for an energetically strong N-terminal basic/hydrophobic motif (epitope 1). However, as the need for affinity from the PY motif decreases and as more binding energy is provided by the two other motifs, sequence tolerance relaxes. The problem of multiple motifs with varying sequence tolerances seems very complex, but the relatively small size of each motif and energetic independence of the motifs allow the problem to be divided into manageable pieces. Our current inability to identify sequences of individual epitopes that are energetically strong may contribute to the 55% accuracy for bioinformatics-derived potential yeast bPY/L-NLS binding to Kap104p. For example, individual epitopes in bioinformatics-derived sequences that did not bind Kap104p may be energetically weak and thus did not provide sufficient binding energy when combined.

First, the range of energies for PY-NLSs that are import-competent in vivo (and to what degree) will need to be determined. The range of suitable binding energies likely will vary depending on cellular concentrations of substrates but should not be unbounded [57]. For example, a designed peptide with a *K*_D_ of 100 pM binds Kapβ2 too tightly for in vivo nuclear import [26], thus providing a high-affinity boundary for Kapβ2 import. Second, binding energies of putative PY-NLSs will need to be predicted. Unfortunately, the accuracy of calculating binding affinity for protein–small molecule interaction is still questionable, and predictions of binding energies for protein–protein interactions are even further behind [58]. Our studies here suggest that we can get around this problem by handling each epitope independently and then combining them to assess for functional NLSs. We may use computational alanine-scanning mutagenesis [59] to predict binding energy differences for each of the three PY-NLS linear epitopes and then empirically determine combinations that are functional. Such predictions could be tested against a future experimental thermodynamic database obtained from the initial predicted PY-NLSs [16], and the method was refined iteratively. Binding energy calculation remains problematic. We expect that prevalent sequence- and physical-characteristics-based bioinformatics methods are limited to successful prediction of potential NLSs with at least one energetically strong linear epitope but will miss those composed of multiple weak or intermediate epitopes. A computational method that combines bioinformatics, structural modeling, and prediction of binding energies may be a solution. Many more Kapβ2–NLS structures will be necessary to expand a structural database to facilitate modeling interactions of new sequences by homology modeling and/or physical energy function-based predictions of protein–protein interactions [60–62].

### Conclusions

PY-NLSs are very diverse in sequence and structure and thus cannot be described sufficiently by their weak consensus motifs. Instead, PY-NLSs are described by a collection of weak physical rules that also include requirements for intrinsic structural disorder and overall positive charge [16]. Here, we examined the energetic organization of PY-NLSs through mutagenic and thermodynamic analyses of these signals in yeast. These studies have revealed physical properties that govern the binding affinity of this variable signal. The PY-NLS is a modular signal composed of three spatially distinct but structurally conserved linear epitopes that can be represented by a series of sequence patterns. Although each linear epitope can accommodate substantial sequence diversity, we have begun to define limits for each. More importantly, in addition to structural modularity, the three linear epitopes also exhibit energetic modularity. Modular organization of the PY-NLS suggests that the daunting search for these very diverse sequences can be performed in parts. Finally, each linear epitope can contribute very differently to total binding energy in different PY-NLSs, explaining how signal diversity can be achieved through combinatorial mixing of energetically weak and strong motifs while maintaining affinity appropriate for nuclear import function. This collection of physical rules and properties describes how functional determinants of the PY-NLSs are organized and lays a path to decode this diverse and evolvable signal for future genome-wide identification of Kapβ2 import substrates. More generally, many biological recognition processes involve linear recognition motifs with weak and obscure sequence motifs. Physical understanding of how diverse PY-NLS sequences can achieve common biological function may serve as a model for decoding many other weakly conserved and complex signals throughout biology.

## Materials and Methods

### Plasmids and strains.

The *Kap104p* gene (gift from J. Aitchison) was subcloned into the pGEX-Tev vector [34]. Yeast substrate genes were obtained by PCR from a S. cerevisiae genomic DNA library (Novagen) and subcloned into the BamHI and NotI sites of the pGEX-Tev and/or pMAL-Tev vectors [63,64]. Site-directed mutagenesis of Nab2p 201–251 and Hrp1p 494–534 were performed using the QuikChange method (Stratagene) and confirmed by nucleotide sequencing.

Full-length Nab2p and Hrp1p wild-type and mutant genes were subcloned into the SpeI and SmaI sites of a modified pRS415 (*CEN6*, *ARS*, *LEU2*, and *AP^R^*) shuttle vector containing a C-terminal GFP gene [65].

### Cell culture and microscopy.

BY4741 (*MAT* **a** *his3*Δ*1 leu2*Δ*0 met15*Δ*0 ura3*Δ*0*) cells harboring pRS415 plasmids were grown at 30 °C in SC-Leu media to mid-logarithmic phase [66]. Cells were transferred to a 1.5% low-melting-point agarose pad made with SC-leu in a coverslip bottom Wilco dish. Cells were observed on an Olympus IX-81 inverted microscope (60× objective), and images were acquired with a Hamamatsu ORCA-ER camera. All images were analyzed in Image-Pro Plus software (Media Cybernetics). To obtain the N/C ratio, mean fluorescence intensity in a 36-pixel box was measured in the nucleus and cytoplasm for at least 50 cells of each mutant.

### Protein expression and purification.

The GST–Kap104p protein was expressed in Escherichia coli Rosetta(DE3)pLysS cells (Novagen). Cells were lysed using an EmulsiFlex-C5 homogenizer (Avestin). The supernatant was applied to glutathione sepharose (GE Healthcare) and washed extensively with Tris buffer (50 mM Tris, pH 7.5, 100 mM NaCl, 1 mM EDTA, 2 mM DTT, and 20% glycerol). GST–Kap104p was eluted with Tris Buffer plus 20 mM glutathione, pH 8.1. The GST tag was cleaved using 0.5 ml of TEV protease in a total volume of 10 ml and separated from Kap104p using an anion exchange column (GE Healthcare). Kap104p was purified further by gel filtration chromatography in TB buffer (20 mM HEPES, pH 7.3, 110 mM KAc, 2 mM MgAc, 1 mM EGTA, 2 mM DTT, and 20% glycerol).

Yeast substrates and NLSs were expressed in E. coli BL21(DE3). The maltose-binding protein (MBP) NLSs were lysed as above and purified by affinity chromatography using amylose resin (New England Biolabs). After extensive washing with Tris buffer, protein was eluted with Tris buffer plus 10 mM maltose. The protein was purified further by cation exchange chromatography.

The GST substrates were lysed by sonication and immobilized on glutathione sepharose. The protein was washed with TB buffer and left on the beads for binding assays. Human substrates were expressed and purified as previously reported [16].

### Bioinformatics search for new Kap104p substrates.

Potential Kap104p substrates were identified as described in Lee et al. [16]. Sequence patterns Φ_1_-G/A/S-Φ_3_-Φ_4_-X_7–12_-R/K/H-X_2–5_-P-Y/L (Φ_1_ is a hydrophobic residue and Φ_3_ and Φ_4_ are hydrophobic residues or R or K) and K/R-X_0–6_-K/R-X_0–6_-K/R-X_0–6_-K/R-X_2–5_-R/K/H-X_1–5_-PY were used in ScanProsite [40] to screen S. cerevisiae proteins in the UniProtKB/Swiss-Prot database [41].

### Binding assays.

Approximately 30 μg of Kap104p was added to ∼10 μg of GST protein immobilized on 20 μl of glutathione sepharose followed by extensive washes with TB buffer and a second incubation with either buffer or RanGTP (5-fold molar excess). Immobilized proteins were visualized with SDS-PAGE and Coomassie staining.

### Isothermal titration calorimetry.

Affinities of wild-type and mutant MBP–Nab2p NLS and MBP–Hrp1p NLS binding to Kap104p were determined by ITC using a MicroCal Omega VP-ITC calorimeter (MicroCal). Proteins were dialyzed against buffer containing 20 mM Tris, pH 7.5, 100 mM NaCl, 2 mM β-mercaptoethanol, and 10% glycerol. The 90–350 μM MBP-NLS proteins were titrated into a sample cell containing 9–35 μM Kap104p. All ITC experiments were done at 20 °C with 35 rounds of 8-μl injections. Data were plotted and analyzed with a single-site binding model using MicroCal Origin software (version 7.0).

## Supporting Information

Figure S1 Isothermal Titration Calorimetry Measurements of Kap104p Binding to (A) MBP–Hrp1p NLS and (B) MBP–Nab2p NLS. (89 KB PDF)

Figure S2 Binding Assays of Kapβ2 with Immobilized Nab2p NLS and Hrp1p NLS in the Presence and the Absence of RanGTP Bound proteins are stained with Coomassie blue.
(70 KB PDF)

Figure S3 Kap104 Recognizes Basic PL-NLSs (A) The sequences of predicted basic PL motifs in yeast proteins. Basic motifs are shaded in dark gray, and the RX_2–5_PY(L) motif is in bold and underlined.
(B) Experimental validation of predicted bPL substrates. Kap104p is added to immobilized GST–NLSs in the presence or absence of RanGTP. Bound proteins are visualized with Coomassie blue.
(180 KB PDF)

Figure S4 Kap104p Specificity (A) Alignment of Kapβ2 homologs showing residues contacting the hydrophobic motif of hPY-NLS (black asterisks) and the basic motif of bPY-NLS (gray asterisks). Contact residues that differ in Homo sapiens (yellow) and S. cerevisiae (gray) are highlighted.
(B) Sequence identity within HEAT repeats of Kapβ2s from different species. Organisms in the Animalia kingdom are colored in red, Plantae kingdom in black, and the Fungi kingdom in blue. The motifs recognized by the B helix of each HEAT repeat are specified above the graph.
(187 KB PDF)

Table S1 Summary of ITC Data for Kap104p Binding to Additional Hrp1p and Nab2p Mutants (91 KB PDF)

Table S2 Summary of ITC Data for Mutations of the PY Motif of Hrp1p (78 KB PDF)

Table S3 Summary of ITC Data for Mutations of the PL Motif of Nab2p (82 KB PDF)

Text S1 Differences in Interface Residues between Kap104p and Kapβ2 (10 KB PDF)

Text S2 Prediction of Kapβ2 Homologs That Recognize hPY-NLSs (79 KB PDF)

### Accession Numbers

Accession numbers for genes mentioned in this paper from the National Center for Biotechnology Information (http://www.ncbi.nlm.nih.gov) are: *Bbp1p* (855820), *Clg1p* (852657), *hnRNP A1* (3178), *Hrp1p* (853997), *Kap104p* (852305), *Kap*β*2* (3842), *Nab2p* (852755), *Nam8p* (856486), *Pos5p* (855913), *Rml2p* (856660), *Sin3p* (854158), *Sko1p* (855554), *Snp1p* (854749), *TAP* (10482), and *Tfg2p* (852888).

## Abbreviations

hnRNP: heterogeneous nuclear ribonucleoprotein
ITC: Isothermal titration calorimetry
Kap: karyopherin
NLSs: nuclear localization signals
NESs: nuclear export signals
PY-NLSs: proline–tyrosine nuclear localization signals
