# Supplementary material for: Modular Organization and Combinatorial Energetics of Proline–Tyrosine Nuclear Localization Signals
Source: PLoS Biol. 2008 Jun 3;6(6):e137. doi: 10.1371/journal.pbio.0060137 (PMC2408616; doi:10.1371/journal.pbio.0060137)
Supplement: Supplementary file 1 [file pbio.0060137.sd001.doc]

**Differences in interface residues between Kap104p and Kapβ2**

Nineteen of forty residues on the Kapβ2-NLS interface are different in yeast and human. Most of these residues are located at interfaces for the N-terminal motifs (H15-H17) and in the linkers between the N- and C-terminal motifs (H12-H14). Interestingly, in the N-terminal motif interfaces, all residues that differ between yeast and human contact the hydrophobic motif in hnRNP A1 but not the basic residues in hnRNP M (Figures 3A, S4A and S4B). These include Kapβ2 residues I722, S723, N726, E734, T766 and I773 that are replaced with T, P, I, L, S and V, respectively, in yeast. Kapβ2 residues I722, S723 and T766 contact substrate M276 in the FGPM hydrophobic motif of hnRNP A1 (Figure S4A), and changes in yeast may compromise interaction with a hydrophobic residue. Kapβ2 residue N726 makes a polar contact with the backbone carbonyl of G274 in hnRNP A1, and the loss of backbone constraints upon the change to isoleucine may disrupt hydrophobic motif recognition. Kapβ2 residues E653, K696, E734, R776 and E809 form an extensive charged network that appears to position the aliphatic portions of R776 and E809 for hydrophobic contacts with the F273 ring in hnRNP A1, while the Cδ of Kapβ2 I773 contacts Cβ of hnRNP A1 F273. In yeast, these contacts are lost as the charged network is disrupted and Cδ of I773 is no longer available for hydrophobic interaction in the yeast valine residue. In contrast, of the many Kapβ2 residues that contact basic sidechains in bPY-NLS, only E653 is different in yeast (Figure S4B). In fact, several amino acid differences at the periphery of the NLS site in yeast Kap104p increase electronegativity, thus supporting bPY-NLS recognition.
