# Supplementary material for: Modular Organization and Combinatorial Energetics of Proline–Tyrosine Nuclear Localization Signals
Source: PLoS Biol. 2008 Jun 3;6(6):e137. doi: 10.1371/journal.pbio.0060137 (PMC2408616; doi:10.1371/journal.pbio.0060137)
Supplement: Supplementary file 2 [file pbio.0060137.sd002.doc]

**Prediction of Kapβ2 homologs that recognize hPY-NLSs**

Based on the sequence alignment of Kapβ2 homologs, we can track interface residues (Figures S4A) and potential overall helical similarities (Figure S4B) at the N-terminal hydrophobic motif interfaces in different organisms and use this information to predict species that recognize hPY-NLS. In higher eukaryotes of the Animalia Kingdom such as *X. laevis* and *D. melanogaster*, all seven Kapβ2 residues that contact the hydrophobic motif are conserved (Figure S4A) and overall sequence identities of H16-H17 are >60% (Figure S4B), suggesting recognition of the hydrophobic PY-NLSs. Organisms in the Plantae Kingdom such as *A. thaliana* and *O. sativa* have few amino acid substitutions (Figure S4A), maintain overall sequence similarities of ~50% in this region (Figure S4B), and are likely to recognize the hydrophobic motif as well. However, Kapβ2 sequences diverge in the Fungi Kingdom (Figure S4A) and overall sequence identities in H16-H17 dip below 30% (Figure S4B), suggesting changes in helical orientations; most fungi Kapβ2s will likely not recognize hPY-NLSs.
