# Supplementary material for: Modular Organization and Combinatorial Energetics of Proline–Tyrosine Nuclear Localization Signals
Source: PLoS Biol. 2008 Jun 3;6(6):e137. doi: 10.1371/journal.pbio.0060137 (PMC2408616; doi:10.1371/journal.pbio.0060137)
Supplement: Supplementary file 3 [file pbio.0060137.sg001.doc]

**Figure S1.** Isothermal titration calorimetry (ITC) measurements of Kap104p binding to MBP-Hrp1p-NLS (*a*) and MBP-Nab2p-NLS (*b*).
