# Supplementary material for: Modular Organization and Combinatorial Energetics of Proline–Tyrosine Nuclear Localization Signals
Source: PLoS Biol. 2008 Jun 3;6(6):e137. doi: 10.1371/journal.pbio.0060137 (PMC2408616; doi:10.1371/journal.pbio.0060137)
Supplement: Supplementary file 4 [file pbio.0060137.sg002.doc]

**Figure S2.** Binding assays of Kapβ2 with immobilized Nab2p-NLS and Hrp1p-NLS in the presence and absence of RanGTP. Bound proteins are Coomassie blue-stained.
