# Supplementary material for: Modular Organization and Combinatorial Energetics of Proline–Tyrosine Nuclear Localization Signals
Source: PLoS Biol. 2008 Jun 3;6(6):e137. doi: 10.1371/journal.pbio.0060137 (PMC2408616; doi:10.1371/journal.pbio.0060137)
Supplement: Supplementary file 5 [file pbio.0060137.sg003.doc]

**Figure S3.** Kap104 recognizes potential basic-PL-NLSs. *(a)* The sequences of predicted basic-PL motifs in yeast proteins. Basic motifs are shaded in dark grey and the Rx_2-5_PY(L) motif is in bold and underlined. *(b)* Experimental testing of predicted bPL substrates. Kap104p is added to immobilized GST-NLSs in the presence or absence of RanGTP. Bound proteins are visualized with Coomassie blue. Faint bands at ~ 70kD are likely heat shock protein contaminants.
