# Supplementary material for: Modular Organization and Combinatorial Energetics of Proline–Tyrosine Nuclear Localization Signals
Source: PLoS Biol. 2008 Jun 3;6(6):e137. doi: 10.1371/journal.pbio.0060137 (PMC2408616; doi:10.1371/journal.pbio.0060137)
Supplement: Supplementary file 6 [file pbio.0060137.sg004.doc]

**Figure S4.** Kap104p specificity. (*a*) Alignment of Kapβ2 homologs showing residues contacting the hydrophobic motif of hPY-NLS (black asterisks) and the basic motif of bPY-NLS (grey asterisks). Contact residues that differ in *H. sapiens* (yellow) and *S. cerevisiae* (grey) are highlighted. (*b*) Sequence identity within HEAT repeats of Kapβ2s from different species. Organisms in the Animalia Kingdom are colored in red, Plantae Kingdom in black, and the Fungi Kingdom in blue. The motifs recognized by the B-helix of each HEAT repeat are specified above the graph.
