# Supplementary material for: Modular Organization and Combinatorial Energetics of Proline–Tyrosine Nuclear Localization Signals
Source: PLoS Biol. 2008 Jun 3;6(6):e137. doi: 10.1371/journal.pbio.0060137 (PMC2408616; doi:10.1371/journal.pbio.0060137)
Supplement: Supplementary file 7 [file pbio.0060137.st001.doc]

**Table S1**

*Summary of ITC data for Kap104p binding to additional Hrp1p and Nab2p mutants*

| *Hrp1p* | *K_D_*^a^  *(nM)* | *∆G*^b^  *(kcal/mol)* | *∆H*  *(kcal/mol)* | *T∆S*^c^  *(kcal/mol/K)* | *∆∆G*^d^  *(kcal/mol)* | *K_Dmutant_/K_Dwildtype_* |
| --- | --- | --- | --- | --- | --- | --- |
| N510A, H511A^e^ | 4±2 | -11.3±0.3 | -13.8±0.2 | -2.6±0.3 | -1.2±0.4 | 0.1±0.8 |
| R512A | 19±4 | -10.3±0.1 | -10.5±1.0 | 0.2±1.0 | -0.3±0.1 | 0.6±0.1 |
| R513A^e^ | 11±5 | -10.6±0.2 | -10.0±0.2 | 0.6±0.3 | -0.6±0.4 | 0.3±0.7 |
| R516A^e^ | 7±1 | -10.9±0.1 | -10.7±0.08 | 0.2±0.1 | -0.9±0.3 | 0.2±0.4 |
| N526A, N527A^e^ | 17±9 | -10.4±0.3 | -11.2±0.3 | -0.8±0.4 | -0.3±0.4 | 0.5±0.8 |
| H530A | 55±12 | -9.7±0.1 | -9.0±1.0 | 0.7±1.1 | 0.3±0.1 | 1.7±0.4 |
| *Nab2p* | *K_D_*^a^  *(nM)* | *∆G*^b^  *(kcal/mol)* | *∆H*  *(kcal/mol)* | *T∆S*^c^  *(kcal/mol/K)* | *∆∆G*^d^  *(kcal/mol)* | *K_Dmutant_/K_Dwildtype_* |
| V214A | 14±8 | -10.5±0.3 | -13.1±0.3 | -2.5±0.4 | -0.6±0.4 | 0.4±0.9 |
| K216A | 22±4 | -10.2±0.1 | -12.9±1.3 | -2.6±1.4 | -0.3±0.1 | 0.6±0.1 |
| R218A | 28±6 | -10.1±0.1 | -11.9±0.5 | -1.8±0.4 | -0.2±0.1 | 0.8±0.2 |
| R219A | 26±4 | -10.2±0.07 | -12.0±0.4 | -1.9±0.3 | -0.2±0.08 | 0.7±0.1 |
| R222A | 27±10 | -10.1±0.2 | -10.7±0.9 | -0.6±0.6 | -0.2±0.2 | 0.7±0.3 |
| R226A | 18±17 | -10.5±0.7 | -11.8±0.3 | -1.2±1.0 | -0.6±0.7 | 0.5±0.5 |
| R229A | 12±10 | -10.7±0.6 | -11.7±0.7 | -1.0±0.2 | -0.8±0.5 | 0.3±0.3 |
| N230A, N231A, N232A | 14±2 | -10.5±0.1 | -12.5±1.5 | -2.0±1.6 | -0.4±0.3 | 0.4±0.006 |
| N237A^e^ | 30±7 | -10.0±0.1 | -10.2±0.1 | -0.2±0.2 | -0.1±0.3 | 0.8±0.5 |
| P238A | 17±4 | -10.4±0.1 | -6.6±0.03 | 3.7±0.09 | -0.5±0.1 | 0.5±0.1 |

^a^ stoichiometry = 0.9-1.1; ^b^ ΔG = -RTlnK_a_; ^c^ TΔS = (ΔH-ΔG); ^d^ ΔΔG = -RTln(K_d_wt_/K_d_mut_); n.d., not detectable;

All binding experiments were performed 2-4 times (± SD) excluding those marked (^e^), which were measured once.
