# Supplementary material for: Modular Organization and Combinatorial Energetics of Proline–Tyrosine Nuclear Localization Signals
Source: PLoS Biol. 2008 Jun 3;6(6):e137. doi: 10.1371/journal.pbio.0060137 (PMC2408616; doi:10.1371/journal.pbio.0060137)
Supplement: Supplementary file 8 [file pbio.0060137.st002.doc]

**Table S2**

*Summary of ITC data for mutations of the PY motif of Hrp1p*

| *Hrp1p*^e^ ^f^ | *K_D_*^a^  *(nM)* | *∆G*^b^  *(kcal/mol)* | *∆H*  *(kcal/mol)* | *T∆S*^c^  *(kcal/mol/K)* | *∆∆G*^d^  *(kcal/mol)* | *K_Dmutant_/K_Dwt_* |
| --- | --- | --- | --- | --- | --- | --- |
| ^531^PY^532^(wildtype) | 32±16 | -10.1±0.3 | -10.0±0.5 | 0.09±0.7 | — | — |
| ^531^PY^532^/PM | 2237±400 | -7.6±0.1 | -4.4±0.2 | 3.2±0.2 | 2.5±0.3 | 69.9±0.4 |
| ^531^PY^532^/PF | 116±30 | -9.2±0.2 | -8.0±0.2 | 1.2±0.2 | 0.8±0.3 | 3.6±0.5 |
| ^531^PY^532^/PH | 1002±350 | -8.0±0.2 | -8.8±0.7 | -0.7±0.7 | 2.0±0.4 | 27.1±0.6 |

^a^ stoichiometry = 0.9-1.1; ^b^ ΔG = -RTlnK_a_; ^c^ TΔS = (ΔH-ΔG); ^d^ ΔΔG = -RTln(K_d_wt_/K_d_mut_); ^e^ single experiment for each mutant (errors from curve fitting); ^f^ No heat was detected when Y532 was mutated to amino acids G, A, C, S, T, D, E, N, Q, K, R, V, L, I, P or W.
