# Supplementary material for: Modular Organization and Combinatorial Energetics of Proline–Tyrosine Nuclear Localization Signals
Source: PLoS Biol. 2008 Jun 3;6(6):e137. doi: 10.1371/journal.pbio.0060137 (PMC2408616; doi:10.1371/journal.pbio.0060137)
Supplement: Supplementary file 9 [file pbio.0060137.st003.doc]

**Table S3**

*Summary of ITC data for mutations of the PL motif of Nab2p*

| *Nab2p* | *K_D_*^a^  *(nM)* | *∆G*^b^  *(kcal/mol)* | *∆H*  *(kcal/mol)* | *T∆S*^c^  *(cal/mol/K)* | *∆∆G*^d^  *(kcal/mol)* | *K_Dmutant_/K_Dwt_* |
| --- | --- | --- | --- | --- | --- | --- |
| ^238^PL^239^ (wt)^e^ | 37±20 | -10.0±0.3 | -11.3±0.5 | -1.3±0.6 | — | — |
| ^238^PL^239^/PG | 99±25 | -9.4±0.1 | -10.4±0.2 | -1.1±0.3 | 0.6±0.3 | 2.7±0.5 |
| ^238^PL^239^/PA^e^ | 26±9 | -10.2±0.2 | -9.7±0.9 | 0.5±1.1 | -0.2±0.2 | 0.7±0.3 |
| ^238^PL^239^/PC | 32±11 | -10.0±0.2 | -10.6±0.2 | -0.6±0.3 | -0.08±0.3 | 0.9±0.6 |
| ^238^PL^239^/PS | 27±16 | -9.6±0.3 | -13.6±0.5 | -4.0±0.6 | -0.2±0.4 | 0.7±0.9 |
| ^238^PL^239^/PT | 15±6 | -10.5±0.2 | -11.0±0.2 | -0.6±0.3 | -0.5±0.4 | 0.4±0.7 |
| ^238^PL^239^/PD | 412±77 | -8.5±0.1 | -7.6±0.2 | 1.0±0.2 | 1.4±0.3 | 11.1±0.5 |
| ^238^PL^239^/PE | 253±54 | -8.8±0.1 | -5.3±0.2 | 3.5±0.2 | 1.1±0.3 | 6.8±0.5 |
| ^238^PL^239^/PN | 61±32 | -9.6±0.3 | -11.3±0.4 | -1.7±0.5 | 0.3±0.4 | 1.7±0.8 |
| ^238^PL^239^/PQ | 20±4 | -10.3±0.1 | -11.6±0.1 | -1.3±0.2 | -0.4±0.3 | 0.5±0.5 |
| ^238^PL^239^/PK | 18±5 | -10.3±0.2 | -9.7±0.1 | 0.6±0.2 | -0.4±0.3 | 0.5±0.6 |
| ^238^PL^239^/PR | 24±9 | -10.2±0.2 | -11.8±0.2 | -1.7±0.3 | -0.3±0.3 | 0.7±0.7 |
| ^238^PL^239^/PV | 70±11 | -9.6±0.1 | -10.2±0.1 | -0.6±0.1 | 0.4±0.3 | 1.9±0.5 |
| ^238^PL^239^/PI^e^ | 103±30 | -9.3±0.2 | -8.9±0.5 | 0.4±0.3 | 0.6±0.2 | 2.8±0.8 |
| ^238^PL^239^/PM | 43±7 | -9.8±0.1 | -11.6±0.09 | -1.7±0.1 | 0.09±0.3 | 1.2±0.5 |
| ^238^PL^239^/PF | 44±7 | -9.8±0.1 | -8.3±0.06 | 1.6±0.1 | 0.1±0.3 | 1.2±0.5 |
| ^238^PL^239^/PY | 13±4 | -10.5±0.2 | -15.1±0.1 | -4.6±0.2 | -0.6±0.3 | 0.4±0.6 |
| ^238^PL^239^/PW | 21±9 | -10.3±0.2 | -12.0±0.3 | -1.8±0.4 | -0.3±0.4 | 0.6±0.8 |
| ^238^PL^239^/PH | 41±7 | -9.9±0.1 | -9.7±0.07 | 0.1±0.1 | 0.06±0.3 | 1.1±0.5 |
| ^238^PL^239^/PP | 159±26 | -9.1±0.1 | -6.9±0.1 | 2.2±0.2 | 0.8±0.3 | 4.3±0.5 |

^a^ stoichiometry = 0.8-1.1; ^b^ ΔG = -RTlnK_a_; ^c^ TΔS = (ΔH-ΔG); ^d^ ΔΔG = -RTln(K_d_wt_/K_d_mut_); ^e^ At least two binding experiments were performed (±SD). All other experiments were performed once (errors from curve fitting).
